# Superficial ulnar artery identification with vascular Doppler ultrasound: a cross-sectional study

**DOI:** 10.1590/1677-5449.202500642

**Published:** 2026-03-23

**Authors:** Mariana Jordão França, Guilherme Francisco Cé, Solena Ziemer Kusma Fidalski, Graciliano José França

**Affiliations:** 1 Universidade Positivo – UP, Curitiba, PR, Brasil.; 2 Universidade Federal do Paraná – UFPR, Curitiba, PR, Brasil.

**Keywords:** ultrasonography, Doppler, ulnar artery, anatomical variation

## Abstract

**Background:**

The ulnar artery normally follows a deep and medial course beneath the flexor digitorum profundus muscle. Anatomical variants in the upper limb vasculature are relatively common and may result from disruptions in angiogenesis or vasculogenesis. One example of such anatomic variants is presence of a superficial ulnar artery, which follows an atypical path superficial to the flexor digitorum profundus muscle. This variant is clinically important and can be diagnosed using vascular Doppler ultrasound.

**Objectives:**

To determine the incidence of the superficial ulnar artery using vascular Doppler ultrasound and to compare these findings with data from cadaveric studies.

**Methods:**

A cross-sectional study was conducted, examining 314 upper limbs from 157 volunteers using vascular Doppler ultrasound.

**Results:**

The superficial ulnar artery was detected in 2.5% of the participants. This incidence aligns with previous cadaveric studies, in which reported prevalence rates range from 0.7% to 9.4%. Being aware of this variation is of fundamental importance in procedures such as peripheral venipuncture, vein cannulation, forearm flaps, and emergency surgeries of the upper limb.

**Conclusions:**

The superficial ulnar artery is a relatively frequent anatomical variant of the upper limb. The 2.5% incidence observed in this study is consistent with previously published cadaveric studies.

## INTRODUCTION

According to Moore et al.^1^, the arterial supply to the upper limbs starts at the subclavian artery, which originates from the aortic arch on the left and the brachiocephalic trunk on the right. The subclavian artery becomes the axillary artery at the lateral border of the first rib. The axillary artery has three parts, divided by the pectoralis minor muscle: the first part gives off the superior thoracic artery; the second gives origin to the thoracoacromial artery and the lateral thoracic artery; and, finally, the third part gives rise to the subscapular artery, the anterior humeral circumflex artery, and the posterior humeral circumflex artery. After the inferior border of the teres major muscle, the axillary artery becomes the brachial artery. The brachial artery ends at the cubital fossa, at the neck of the radius, below the bicipital aponeurosis, where it branches into the radial and ulnar arteries. The radial artery follows a lateral and superficial path and can be palpated medial of the styloid process of the radius. It is responsible for forming the principal branch of the deep palmar arch, in addition to giving off a branch that contributes to formation of the superficial palmar arch. In turn, the ulnar artery is the largest terminal branch of the division of the brachial artery and is located anterior to the head of the ulna, coursing medial and deep to the flexor digitorum profundus. This artery originates the common interosseous artery, which branches into the posterior and anterior interosseous arteries, and then the flexor retinaculum of the wrist, forming the main part of the superficial palmar arch and contributing to formation of the deep palmar arch.^1^

Anatomic variations of the arteries of the upper limb are common and can occur because of incomplete development, fusion of arteries that are usually separate, or persistence of arteries that are normally eliminated during development, but remain patent.^2^ One such variant is presence of a superficial ulnar artery, in which the ulnar artery follows a path superficial to the flexor muscles of the forearm. It is estimated that the rate of occurrence of cases of this anomaly ranges from 0.7 to 9.4%, being more common in the right upper limb.^3^ The prevalence of bilateral presence of this variation ranges from 0.01 to 0.62%.^4^

Diagnosis of anatomic variations, such as the superficial ulnar artery, is important for surgeons, clinicians, and nursing professionals. Doppler vascular ultrasound is a diagnostic method for detection of anatomic anomalies that is accessible, rapid, noninvasive, and inexpensive.

## OBJECTIVES

The objective of the present study was to examine the right and left ulnar arteries of 157 volunteers using Doppler vascular ultrasound to detect presence of anatomic variants of the superficial ulnar artery and to compare the results with percentages reported in cadaveric studies.

## METHODS

This was a non-interventional cross-sectional study involving examination of 157 patients with Doppler vascular ultrasound, using a Sonosite Titan ultrasound unit and a linear transducer with a frequency range of 5-10 MHz. The study participants were volunteers, enrolling in the sample spontaneously in response to adverts about the study released by the researchers. The sample size calculation was therefore based on the confidence interval of a proportion. This calculation determines the prevalence of a characteristic in the population. The confidence level was 95% and the margin of error was 5%. The estimated proportion in the population was 9.4%.^3^ The calculation indicated a sample of 131 people, totaling 157 people after allowing for a 20% margin of error. The study enrolled 100% of this sample size.

Examinations were conducted by a vascular surgeon with vascular ultrasonography certification, aided by two medical students, who were responsible for recording the data. Both right and left upper limbs were examined, totaling 314 limbs. With regard to ultrasound, presence or absence of the superficial ulnar artery was assessed by conducting vascular ultrasound examination of the medial forearm. The variables selected for analysis were ulnar artery or superficial ulnar artery in the forearm and age and sex of volunteers. Vascular ultrasound examinations were conducted using B-mode, color mode, and Power Doppler.

Data were collected from volunteers at a university in the city of Curitiba, Paraná, Brazil, during October of 2024. Data were computed sequentially by date and order of examination. Data were tabulated in an Excel spreadsheet, which was used for statistical analysis. The chi-square test was applied and odds ratios were calculated. P values < 0.05 were considered significant. The project followed the Strengthening the Reporting of Observational Studies in Epidemiology guidelines for cross-sectional studies.

All patients signed a free and informed consent form. The study was approved by the Ethics Committee in April 2024, with Ethics Appraisal Submission Certificate number 78551324.6.0000.0093 and substantiated opinion number 6.765.516. The inclusion criterion was volunteer participants of both sexes. Participants were excluded if it was only possible to examine one of their upper limbs, to avoid including unilateral data.

## RESULTS

**Figure 1:**
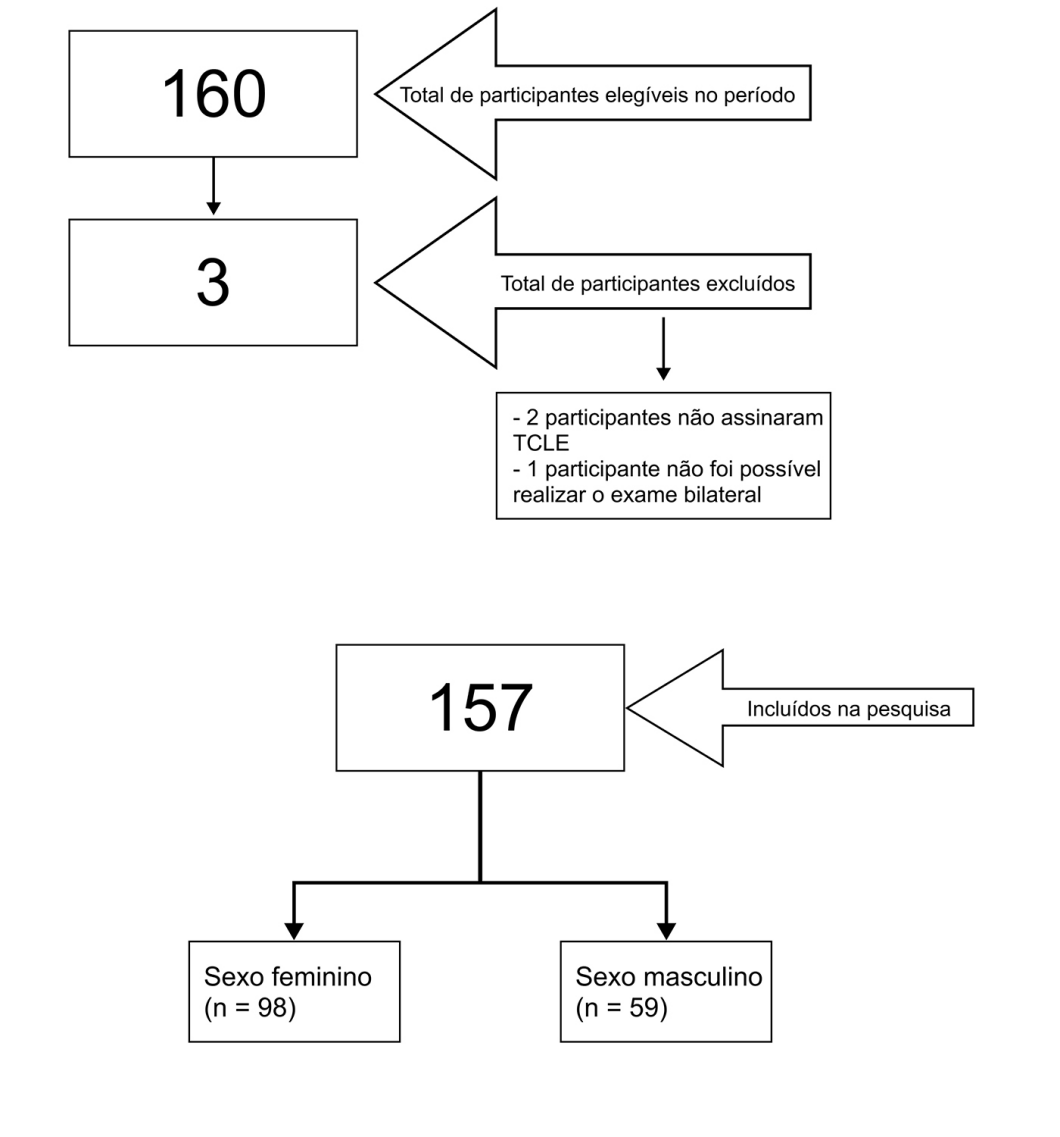
Flow diagram illustrating enrollment of participants on the study.

A total of 160 volunteers were considered eligible. Three people were excluded according to the preestablished exclusion criteria (Figure 1). The sample comprised 157 volunteers who were examined bilaterally during October of 2024 using Doppler vascular ultrasound (Table 1). Among the data collected from 314 upper limbs, five limbs had a superficial ulnar artery (2.5% of the participants).

**Table 1:** Description of the characteristics of the study participants (n = 157).

**Characteristic**	**n**
**%**	
**Age brackets (years)**	
17-24	136
86.6	
25-30	12
7.6	
31-40	7
4.5	
41-50	2
1.3	
**Sex**	
Female	98
62.4	
Male	59
37.6	

Source: The authors (2025).

**Figure 2:**
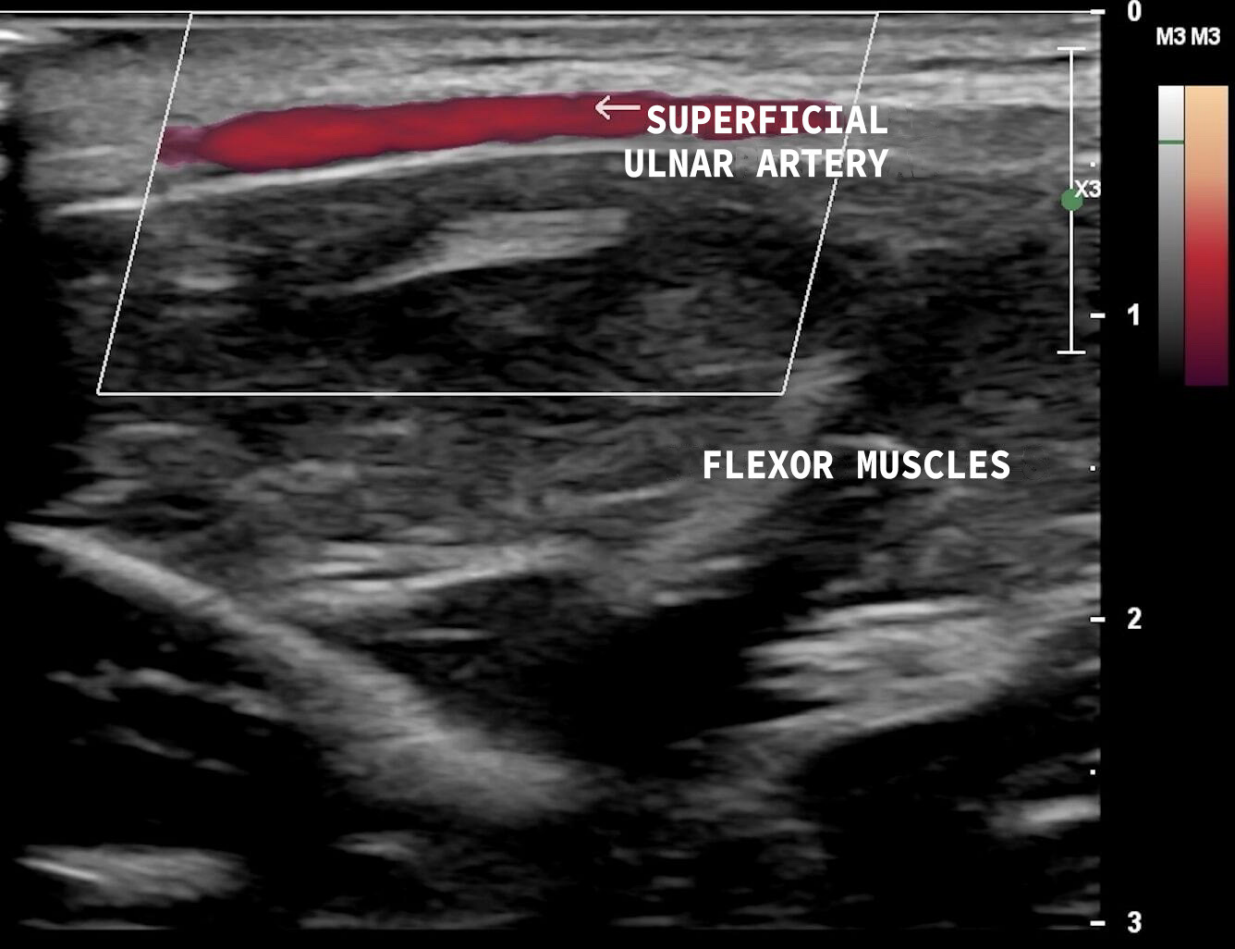
Vascular ultrasound, longitudinal view, with superficial ulnar artery anatomic variant present, coursing above the flexor muscles of the forearm.

**Figure 3:**
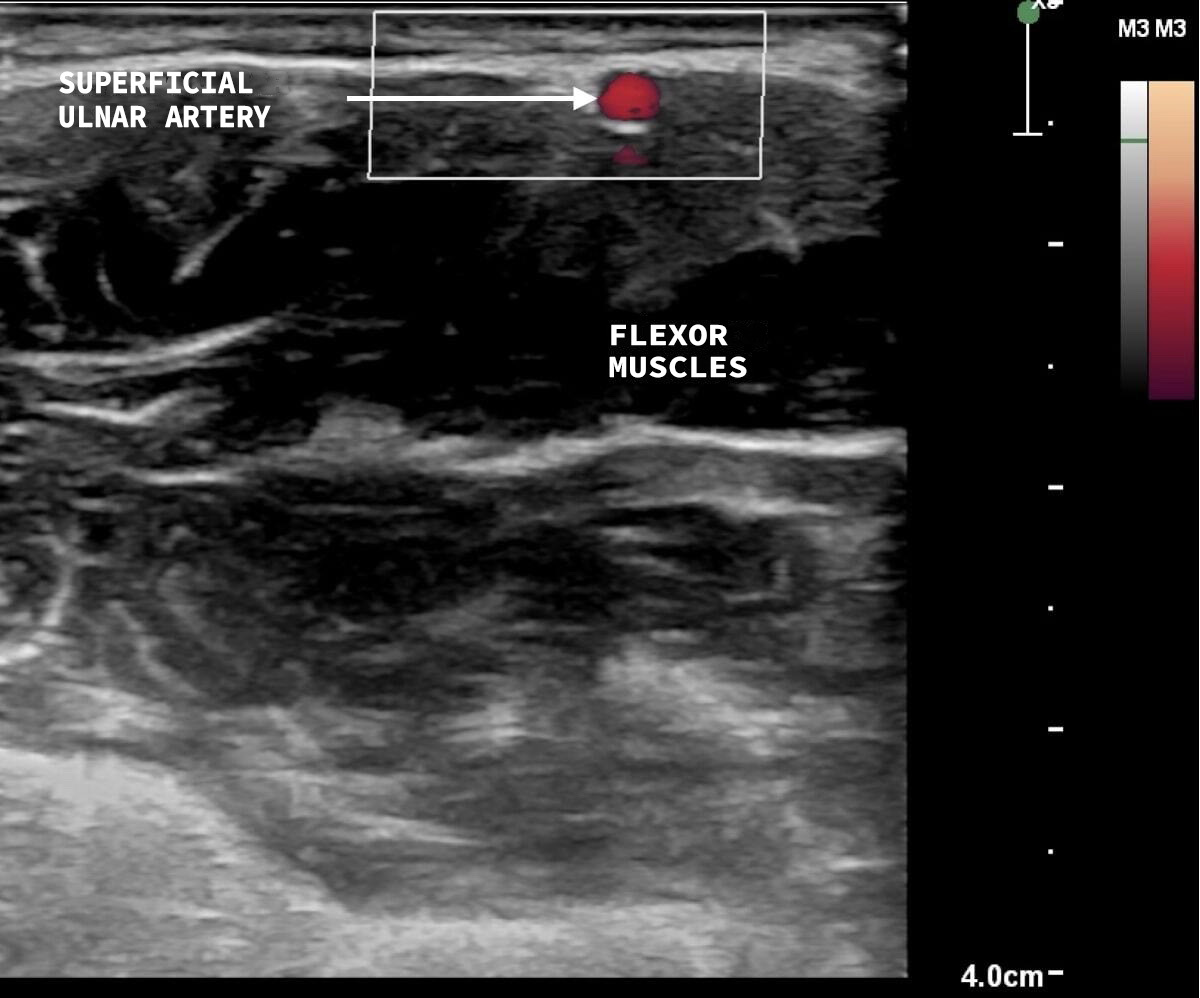
Vascular ultrasound, transverse view, with superficial ulnar artery anatomic variant present, coursing above the flexor muscles of the forearm.

**Figure 4:**
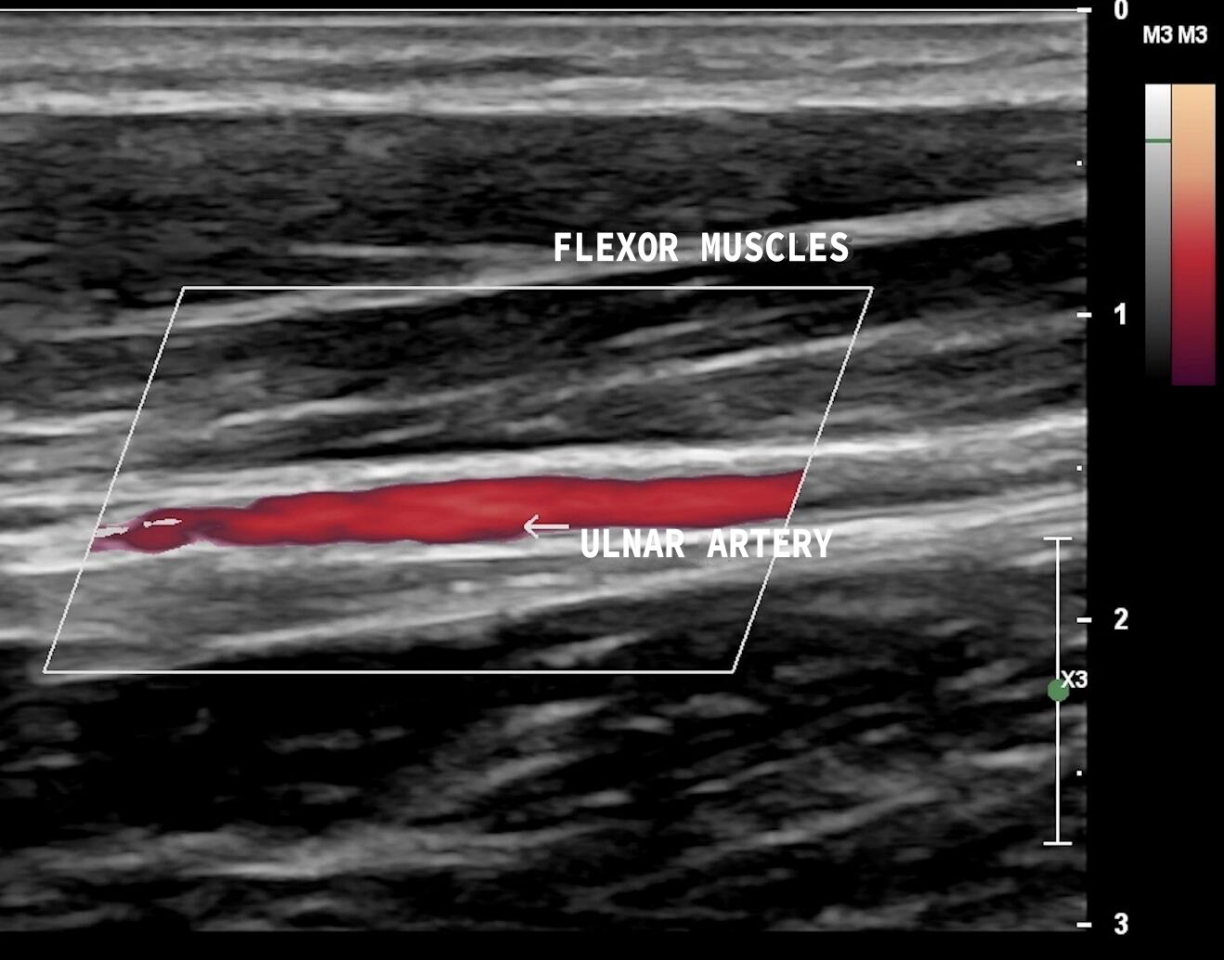
Vascular ultrasound, longitudinal view, with the ulnar artery following its normal path, coursing below the flexor muscles of the forearm.

**Figure 5:**
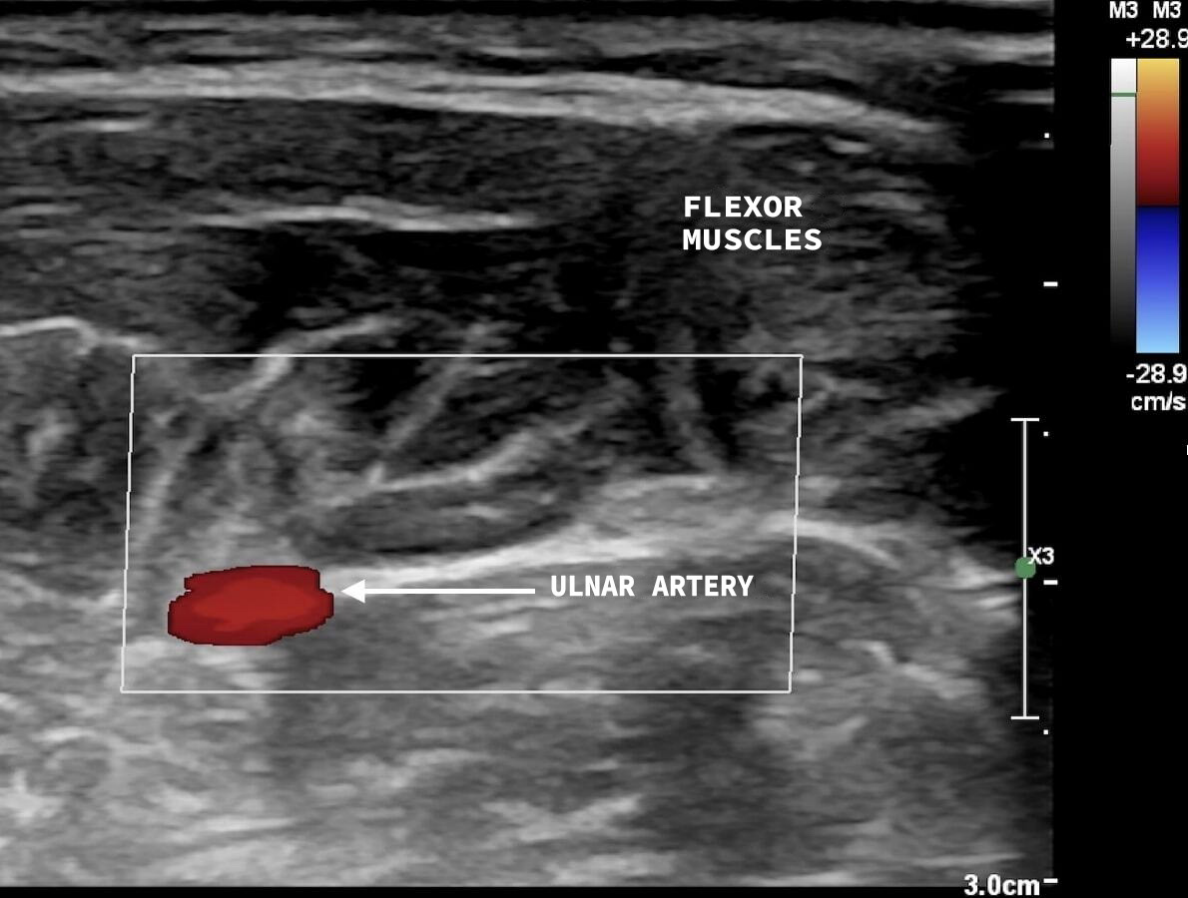
Vascular ultrasound, transverse view, with the ulnar artery following its normal path, coursing below the flexor muscles of the forearm.

The variables studied were superficial ulnar artery, i.e., anatomic variant present (Figures 2 and 3), and ulnar artery, anatomic variant absent (Figures 4 and 5). Where the superficial ulnar artery was present, it was noted whether the variant was present bilaterally, in the right upper limb only, or in the left upper limb only (Table 2). The variant was more common in the right upper limb (observed in three volunteers) and the variant was bilateral in one participant.

**Table 2:** Occurrence of the ulnar artery anatomic variant.

**Characteristic**	**n**
**%**	
**Ulnar artery**	
Anatomic variant absent	153
97.5	
Anatomic variant present	4
2.5	
Bilateral superficial ulnar artery	1
0.6	
Superficial ulnar artery in the right upper limb only	3
1.9	
Superficial ulnar artery in the left upper limb only	0
0	

Source: The authors (2025).

A statistical analysis was conducted of the data collected. The chi-square test (Table 3) showed that although all superficial ulnar artery variants were found in females, this did not constitute a significant difference between females and males for presence of a superficial ulnar artery, since the p value was 0.116, with a 95% confidence level.

**Table 3:** Inferential analysis of the association between ulnar artery anatomic variant and sex.

**Sex Fisher’s exact test**	**Ulnar artery anatomic variant present (%) P value**	**Ulnar artery anatomic variant absent (%)**
Female	4 (4.1%)	94 (95.9%)
0.298	0.116^1^	
Male	0 (0.0%)	59 (100.0%)

1 Chi-square test

Source: The authors (2025).

## DISCUSSION

The superficial ulnar artery is a variant that has been well described in the cadaveric study literature, with prevalence ranging from 0.7 to 9.4% and higher prevalence in the right upper limb.^3^ A cadaveric study by Dartnell et al.,^5^ involving 95 cadavers, reported a 4.2% prevalence of superficial ulnar artery, with 75.0% of cases observed in the right upper limb. A cadaveric study by Funk et al.^6^ concluded that 2.5% of 204 cadavers had the superficial ulnar artery variant. Bhat et al.^7^ report that the superficial ulnar artery may have the usual brachial artery origin or may have a high origin from the axillary artery. In cases with origin from the axillary artery, the superficial ulnar artery may be responsible for arterial supply to the biceps brachii muscle.

The superficial ulnar artery is an anatomic variant of great clinical importance because it courses above the flexor digitorum profundus. Its path superficial of the flexor muscles of the forearm makes the artery more susceptible to bleeding and trauma.^8^ It may be inadvertently injured during peripheral venous punctures attempted for infusion of medications or saline or to draw blood for laboratory tests. The superficial ulnar artery is most likely to be damaged when attempting venous puncture of the median cubital vein or the basilic vein of the forearm which both course medially, in common with the artery.^9^ Pseudoaneurysm formation may occur if the artery is injured inadvertently.^10^ Moreover, iatrogenic lesions may also occur during cubital fossa vein cannulation attempts.^11^ There is a report in the literature of accidental intra-arterial cannulation in a superficial ulnar artery case.^9^

However, there are also advantages to the presence of a superficial ulnar artery. This variant can make it possible to harvest flaps with vascularization by the ulnar artery rather than the radial artery, as is habitually used. In cases requiring a forearm cutaneous flap (Chinese flap), the ulnar tendons will be less exposed than the radials, contributing to a lower rate of complications. In cases requiring a Chinese flap for areas such as the head and neck, there will be esthetic advantages, because the medial aspect of the forearm has less hair growth. Moreover, the medial aspect is less visible than the lateral aspect of the forearm, offering esthetic advantages in terms of postoperative scarring of the donor area. Finally, there are also advantages in terms of the arterial supply to the hand, since it is estimated that the deep palmar arch (primarily formed by the radial artery) is complete in 97% of the patients, whereas the superficial palmar arch (primarily formed by the ulnar artery) is only complete in 79% of the patients. As such, using a Chinese flap in a patient with a superficial ulnar artery offers esthetic advantages for both donor and recipient areas, a lower risk of complications, and better arterial supply to the hand.^12^

Knowledge of the superficial ulnar artery is essential and extremely important for a range of medical practices, such as surgical procedures involving trauma, flaps for reconstruction, and venous cannulation, and also nursing procedures, such as peripheral venous access. It is very important that these patients themselves know they have this anatomic variant, to be able to inform medical teams and avoid incidental arterial puncture during peripheral venous access or cannulation, with the option to use the contralateral limb in unilateral cases. There is also the fact that if a patient has informed a surgeon that they have this variant, there is the possibility of choosing an ulnar flap rather than a radial flap.

The main limitation of this study is that the authors only examined whether the superficial ulnar artery was present or absent and did not assess its origin. It was not therefore possible to report whether, when present, the artery originated from the brachial artery or the axillary artery. This limitation was due to practical conditions during data collection: these examinations were performed during a week of scientific events held at the university and volunteers were examined with their shirts raised above the cubital fossa, restricting access to the proximal arm.

Another limitation is that the sample was selected by convenience and no specific criteria or randomization were employed for selection. There is also the limitation of the Doppler vascular ultrasound technique’s examiner-dependent nature, since assessment and results are dependent on the experience and skill of the professional who conducted the examinations.

It is possible to confirm that the superficial ulnar artery is a frequently-observed variant of the upper limbs. The percentage of individuals in the study sample who had a superficial ulnar artery was 2.5% and the most prevalent location was the right limb, which are results that coincide with cadaveric studies found in the scientific literature.

## Data Availability

Os dados de pesquisa estão disponíveis no corpo do artigo.
