# Supplementary material for: Superficial ulnar artery identification with vascular Doppler ultrasound: a cross-sectional study
Source: J Vasc Bras. 2026 Mar 23;25:e20250064. doi: 10.1590/1677-5449.202500642 (PMC13020968; doi:10.1590/1677-5449.202500642)
Supplement: Translation

# Translation: Avaliação da artéria ulnar superficial através do ultrassom vascular Doppler: um estudo transversal

**DOI:** 10.1590/1677-5449.202500641

**Authors:** Mariana Jordão França, Guilherme Francisco Cé, Solena Ziemer Kusma Fidalski, Graciliano José França

**Affiliations:** 1 Universidade Positivo – UP, Curitiba, PR, Brasil.; 2 Universidade Federal do Paraná – UFPR, Curitiba, PR, Brasil.

**Keywords:** ultrassonografia Doppler, artéria ulnar, variação anatômica

## Abstract

**Contexto:**

A artéria ulnar possui trajeto medial e profundo ao músculo flexor profundo dos dedos. Variações anatômicas em artérias do membro superior são comuns e podem ocorrer por problemas na angiogênese e na vasculogênese, sendo uma dessas variações a presença da artéria ulnar superficial. Nesta variação, a artéria ulnar possui trajeto não usual e superficial ao músculo flexor profundo dos dedos. Essa variação possui importância clínica e pode ser diagnosticada por meio do ultrassom vascular Doppler.

**Objetivos:**

Identificar a incidência da artéria ulnar superficial pelo ultrassom vascular Doppler e comparar os achados com estudos cadavéricos.

**Métodos:**

Estudo transversal em 314 membros superiores de 157 voluntários avaliados pelo ultrassom vascular Doppler.

**Resultados:**

Ao todo, 2,5% dos participantes do nosso estudo apresentaram a variação anatômica da artéria ulnar superficial. Nossa porcentagem está dentro da média encontrada em estudos cadavéricos na literatura, que variou de 0,7 até 9,4%. Ter conhecimento dessa variação é fundamental em procedimentos como punção venosa periférica, canulação venosa, retalhos de antebraço e cirurgias de emergência nos membros superiores.

**Conclusões:**

A artéria ulnar superficial é uma variação frequente no membro superior. O percentual de indivíduos com a presença da artéria ulnar superficial em nosso estudo foi de 2,5%, o que coincide com os dados da literatura de estudos cadavéricos.

## INTRODUÇÃO

De acordo com Moore et al.^1^ , o suprimento arterial dos membros superiores se inicia com a artéria subclávia, a qual tem origem no arco aórtico à esquerda e no tronco braquiocefálico à direita. A artéria subclávia se torna artéria axilar na margem lateral da primeira costela. A artéria axilar possui três partes, divididas pelo músculo peitoral menor: a primeira parte dá origem à artéria torácica superior; a segunda parte origina os ramos da artéria toracoacromial e da artéria torácica lateral; e, por fim, a terceira parte origina a artéria subescapular, a artéria circunflexa anterior do úmero e a artéria circunflexa posterior do úmero. Após a margem inferior do músculo redondo maior, a artéria axilar se torna artéria braquial. A artéria braquial termina na fossa cubital, diante do colo do rádio e sob o revestimento da aponeurose do músculo bíceps braquial, onde se ramifica em artérias radial e ulnar. A artéria radial tem percurso lateral e superficial, sendo palpada medial ao processo estiloide do rádio, e é responsável pela formação do ramo principal do arco palmar profundo, além de emitir um ramo que contribui para a formação do arco palmar superficial. Já a artéria ulnar é o maior ramo terminal da divisão da artéria braquial e situa-se anteriormente à cabeça da ulna, com trajeto medial e profundo ao músculo flexor profundo dos dedos. Essa artéria dá origem à artéria interóssea comum, que se ramifica em artérias interóssea posterior e anterior, e, após o retináculo dos flexores, forma a parte principal do arco palmar superficial e contribui para a formação do arco palmar profundo^1^ .

Variações anatômicas nas artérias do membro superior são comuns e podem ocorrer devido ao desenvolvimento incompleto, à fusão de artérias usualmente separadas ou à persistência de vasos que seriam normalmente obliterados e permanecem pérvios^2^ . Uma dessas variações é a presença da artéria ulnar superficial, em que a artéria ulnar possui trajeto superficial aos músculos flexores do antebraço. Estima-se que a ocorrência de casos dessa anomalia varia entre 0,7 e 9,4%, sendo mais comum a presença no membro superior direito^3^ . A prevalência da variação bilateral é de 0,01 a 0,62%^4^ .

O diagnóstico de variações anatômicas, como a artéria ulnar superficial, possui importância para médicos cirurgiões, clínicos e para profissionais de enfermagem. Um método diagnóstico de fácil acesso, rápido, não invasivo e de baixo custo para detecção de anomalias anatômicas é o ultrassom vascular Doppler.

## OBJETIVOS

O objetivo do presente estudo foi avaliar a artéria ulnar direita e esquerda de 157 voluntários através do ultrassom vascular Doppler, com o intuito de investigar a presença da variação anatômica da artéria ulnar superficial e comparar os dados encontrados em nosso estudo com a porcentagem relatada em estudos cadavéricos.

## MÉTODOS

Trata-se de um estudo transversal e não intervencionista por meio da realização de exames de ultrassom vascular Doppler, com aparelho de ultrassom Sonosite Titan, transdutor linear com frequência de 5-10 MHz, em 157 pacientes. Os participantes da pesquisa eram voluntários, contribuindo para a amostra de forma espontânea após a divulgação do estudo pelos pesquisadores. Assim, o cálculo do tamanho da amostra foi realizado por meio do intervalo de confiança de uma proporção. Esse cálculo determina a prevalência de uma característica da população. O nível de confiança foi de 95% e a margem de erro foi de 5%. A proporção estimada na população foi de 9,4%^3^ . O cálculo apontou uma amostra de 131 pessoas, considerando uma possibilidade de erro de 20%, totalizando 157 pessoas. Nesta pesquisa, atingiu-se 100% da amostra prevista.

Os exames foram realizados por um cirurgião vascular com certificado em ultrassonografia vascular, acompanhado por dois alunos de Medicina, os quais eram responsáveis pelo registro dos dados. A avaliação foi realizada nos membros superiores direito e esquerdo, perfazendo 314 membros examinados. Do ponto de vista ultrassônico, a presença ou ausência da artéria ulnar superficial foi estudada por meio da realização do ultrassom vascular na região medial do antebraço. As variáveis selecionadas foram artéria ulnar ou artéria ulnar superficial no antebraço, sexo e idade dos voluntários. O ultrassom vascular foi realizado por meio do modo B, modo colorido e Power Doppler.

Foram colhidos dados de voluntários em uma universidade na cidade de Curitiba, no Paraná, em outubro de 2024. Os dados foram computados sequencialmente por data e ordem da realização do exame nos participantes. As informações foram tabuladas em planilha no Excel, com as quais foi feita uma análise estatística. Os testes utilizados foram o qui-quadrado e a razão de chances. Valores de p < 0,05 foram considerados significativos. O projeto seguiu as diretrizes *Strengthening the Reporting of Observational Studies in Epidemiology* para estudos seccionais.

Todos os pacientes assinaram o termo de consentimento livre e esclarecido. O estudo foi aprovado pelo Comitê de Ética em abril de 2024, com o número de Certificado de Apresentação de Apreciação Ética 78551324.6.0000.0093 e parecer consubstanciado nº 6.765.516. Os critérios de inclusão foram participantes de ambos os sexos, seguindo o critério de voluntariedade. Foram excluídos participantes dos quais só foi possível a realização do exame de um único membro superior, a fim de evitar a entrada de dados unilaterais.

## RESULTADOS

Um total de 160 voluntários foram considerados elegíveis. Três pessoas foram excluídas conforme os critérios de exclusão previamente definidos (Figura 1). Foram incluídos 157 voluntários que tiveram seus exames bilaterais realizados em outubro de 2024 por meio do ultrassom vascular Doppler (Tabela 1). Dos dados obtidos a partir de 314 membros superiores avaliados, cinco membros apresentaram a presença da artéria ulnar superficial (2,5% dos participantes).

**Figura 1:** Fluxograma dos participantes incluídos no estudo. TCLE = termo de consentimento livre e esclarecido.

**Tabela 1:** Descrição das características dos participantes da pesquisa (n = 157).

**Característica**	**n**
**%**	
**Faixa etária (anos)**	
17-24	136
86,6	
25-30	12
7,6	
31-40	7
4,5	
41-50	2
1,3	
**Sexo**	
Feminino	98
62,4	
Masculino	59
37,6	

Fonte: Autores (2025).

As variáveis estudadas foram artéria ulnar superficial, com variação anatômica (Figuras 2 e 3), e artéria ulnar, sem variação anatômica (Figuras 4 e 5). No caso da presença da artéria ulnar superficial, foi detalhado se a variação ocorreu bilateralmente, no membro superior direito ou no membro superior esquerdo (Tabela 2). A presença da variação foi mais comum no membro superior direito (três voluntários), e a variação bilateral ocorreu em um participante.

**Figura 2:** Ultrassom vascular, corte longitudinal, com a presença da variação anatômica da artéria ulnar superficial, com trajeto acima dos músculos flexores do antebraço.

**Figura 3:** Ultrassom vascular, corte transversal, com a presença da variação anatômica da artéria ulnar superficial, com trajeto acima dos músculos flexores do antebraço.

**Figura 4:** Ultrassom vascular, corte longitudinal, com a artéria ulnar em seu trajeto usual, abaixo dos músculos flexores do antebraço.

**Figura 5:** Ultrassom vascular, corte transversal, com a artéria ulnar em seu trajeto usual, abaixo dos músculos flexores do antebraço.

**Tabela 2:** Ocorrência da variação anatômica da artéria ulnar.

**Característica**	**n**
**%**	
**Artéria ulnar**	
Sem variação anatômica	153
97,5	
Com variação anatômica	4
2,5	
Artéria ulnar superficial bilateral	1
0,6	
Artéria ulnar superficial no membro superior direito	3
1,9	
Artéria ulnar superficial no membro superior esquerdo	0
0	

Fonte: Autores (2025).

Com os dados coletados, foi realizada uma análise estatística. A partir da aplicação do teste do qui-quadrado (Tabela 3), foi possível afirmar que, mesmo com todas as variantes de artéria ulnar superficial sendo encontradas em nossa amostra no sexo feminino, não existiu diferença significativa entre os sexos feminino e masculino para a presença da artéria ulnar superficial, já que o valor de p encontrado foi de 0,116, considerando uma razão de confiabilidade de 95%.

**Tabela 3:** Análise inferencial da associação entre variação anatômica na artéria ulnar por sexo.

**Sexo Teste exato de Fisher**	**Variação anatômica na artéria ulnar (%) Valor de p**	**Sem variação anatômica na artéria ulnar (%)**
Feminino	4 (4,1%)	94 (95,9%)
0,298	0,116^1^	
Masculino	0 (0,0%)	59 (100,0%)

1 Teste do qui-quadrado

Fonte: Autores (2025).

## DISCUSSÃO

A artéria ulnar superficial é uma variação bem descrita na literatura de estudos cadavéricos, que possui prevalência entre 0,7 e 9,4%, sendo o membro superior direito o mais prevalente^3^ . Um estudo cadavérico de Dartnell et al.^5^ , com 95 cadáveres, demonstrou a prevalência da artéria ulnar superficial em 4,2% dos membros superiores, sendo que 75,0% desses casos ocorreram no membro superior direito. A pesquisa cadavérica de Funk et al.^6^ , com 204 cadáveres, concluiu que 2,5% dos membros apresentavam a variação da artéria ulnar superficial^6^ . Bhat et al.^7^ relatam que a artéria ulnar superficial pode ter origem usual da artéria braquial ou até mesmo origem alta a partir da artéria axilar. Nos casos de origem a partir da artéria axilar, a artéria ulnar superficial pode suprir a irrigação arterial do músculo bíceps braquial.

Devido a seu trajeto acima do músculo flexor profundo dos dedos, a artéria ulnar superficial é uma variação anatômica de grande importância clínica. Seu trajeto superficial aos músculos flexores do antebraço deixa a artéria mais suscetível a sangramentos e traumas^8^ . Pode ocorrer lesão inadvertida da artéria em tentativas de punção venosa periférica para infusão de medicamentos, soro ou coleta de exames laboratoriais. A artéria ulnar superficial pode ser lesada principalmente nas tentativas de punção venosa da veia intermédia do cotovelo e da veia basílica do antebraço, que, assim como a artéria, também possuem trajeto medial^9^ . Em casos de lesão inadvertida da artéria, pode ocorrer a formação de pseudoaneurisma^10^ . Além disso, pode ocorrer lesão iatrogênica em tentativas de canulação de veia da fossa cubital^11^ . Foi reportada na literatura canulação intra-arterial acidental em casos de artéria ulnar superficial^9^ .

Por outro lado, existem vantagens da presença da artéria ulnar superficial. Essa variação pode permitir o uso de retalhos com vascularização da artéria ulnar ao invés da artéria radial, que é usada de forma habitual. Nos casos de retalho cutâneo do antebraço (retalho chinês), haverá menor exposição dos tendões ulnares do que dos radiais, o que contribui para uma menor taxa de complicações. Em casos de uso do retalho chinês para áreas como cabeça e pescoço, existem vantagens estéticas, pois a face medial do antebraço possui menor pilificação. Além disso, a face medial é menos visível que a face lateral do antebraço, contribuindo com vantagens estéticas da cicatriz pós-operatória da área doadora. Por fim, existe uma vantagem no suprimento arterial da mão, pois estima-se que o arco palmar profundo (formado principalmente pela artéria radial) é completo em 97% dos pacientes, enquanto o arco palmar superficial (formado principalmente pela artéria ulnar) é completo em apenas 79% dos pacientes. Portanto, utilizar o retalho chinês ulnar em pacientes com artéria ulnar superficial apresenta vantagens estéticas para a área doadora e receptora, menor risco de complicações e garante melhor suprimento arterial para a mão^12^ .

Ter conhecimento a respeito da artéria ulnar superficial é essencial e de extrema importância em diversas práticas médicas, como áreas cirúrgicas que envolvam operações de trauma, retalhos para reconstrução e canulação venosa, além de procedimentos de enfermagem, como acesso venoso periférico. É de grande importância que o paciente tenha conhecimento a respeito da variação anatômica, com o objetivo de informar a equipe e evitar a punção incidental da artéria durante o acesso venoso periférico ou canulação, podendo-se optar pelo membro superior contralateral em casos de variação unilateral. Soma-se a isso o fato de o paciente avisar o cirurgião, podendo-se optar, nos casos de variação, pelo retalho ulnar ao invés do radial.

A principal limitação deste estudo foi o fato de que os autores examinaram apenas a presença ou ausência da artéria ulnar superficial, não sendo avaliada a sua origem. Não foi possível determinar, quando presente, se a artéria era originada da artéria braquial ou da artéria axilar. Essa limitação ocorreu devido às condições práticas durante a coleta de dados: os exames foram realizados durante uma semana de eventos científicos da faculdade, e os voluntários foram avaliados com as blusas levantadas acima da fossa cubital, o que restringiu o acesso à região proximal do braço. Além disso, existe a limitação de a amostra selecionada ser de conveniência, não sendo adotados critérios específicos ou aleatoriedade na seleção. Soma-se a isso a limitação de o ultrassom vascular Doppler ser uma técnica examinador-dependente, pois a avaliação e os resultados podem depender da experiência e habilidade do profissional que realizou os exames.

É possível constatar que a artéria ulnar superficial é uma variante encontrada com frequência nos membros superiores. O percentual de indivíduos com artéria ulnar superficial em nosso estudo foi de 2,5%, e o membro mais prevalente foi o direito, dados que coincidem com estudos cadavéricos encontrados na literatura científica.

## Data Availability

The research data is available in the body of the article.
